# Abstracts from Foreign Journals

**Published:** 1902-04

**Authors:** S. J. J. Harger

**Affiliations:** Philadelphia, Pa.


### ABSTRACTS FROM FOREIGN JOURNALS.

### FRENCH.

Under the Direction of S. J. J. Harger, V.M.D.,
PHILADELPHIA, PA.

Iodine in the Leucocytes. Stassano and Bourcet demonstrated that normal blood contains iodine, and by chemical examination found that this metal was fixed in the white blood cells. —*Comp.-rend. Acad. des Sci., Paris.*

Pyæmia in the Foal. Sohnle, of the national stud of Wurtemberg, after some pathological and bacteriological studies, arranges the diverse manifestations of this affection under one morbid entity. According to him, the infection may take place *in utero* or through the umbilicus by an infected liver; an infected stallion may propagate the disease from mare to mare during coition. The disease has been seen in successive colts from the same mare. According to the records of the stud at Marbach, mares so infected will not become pregnant, abort, or the foal becomes affected a few days after birth, according to the virulence of the germ; the same is said of mares served by a contaminated stallion.

The disease may manifest itself from eight to twenty-four hours after birth. Isolation of the mare is not always a preventive, and Sohnle has found in the genital discharges and in the uterus the microbe which he considers as the specific cause. These are the reasons of infection *in utero*. Alterations of the umbilical cord may be wanting.

The specific microbe is claimed to be a diplococcus encapsulated and found in the tendinous and articular synovials, in the blood, and in the uterine discharges of infected mares. It takes the Gram and aniline stains, grows upon different media, liquefies gelatin in forty-eight hours, and forms colonies of a granular, grayish aspect. It grows rapidly upon potato, forming a thick, moist, brilliant layer of an orange-red color. Under the microscope it appears as an immobile micrococcus provided with a clear, translucent capsule and frequently united " enchâine " of two, three, and four.

The germ is probably a variety of the staphylococcus pyogenes aureus. It is virulent to other animals; 5 c.c. of a bouillon culture injected into the jugular of a horse produces death in three days, with high fever and articular enlargements. At the autopsy lobar pneumonia, suppuration of the articulations of the members and that between the atlas and axis were found. Specific diplococci were abundant.

Preventive measures: 1. Treatment of infected or suspected mares. 2. Disinfection of the stable. 3. Antisepsis of the umbilical cord of the newborn. 4. Disinfection of the penis of the stallion after each service of suspected mares.

The above rules, carefully observed, have succeeded in eradicating the disease from infected stables.—*Monatsch. für präkt. Thierk.*

Antistreptococcus Serum in Anasarca. Anasarca may be a complication of diverse affections in which the streptococcus pyogenes, that of strangles, and the typhic cocobacillus may exist alone or associated in a varied manner. In practice one cannot diagnose the particular kind of anasarca, and, as the serum treatment is harmless, Pécus recommends it in all cases at the beginning. The result may be expected to be positive when it is a question of the streptococcus alone or associated with other bacteria, and negative when the disease is caused by another microbe.—*Ann. de Méd. Vét.*

A New Blister. The following formula was prepared by Rasset, and has given excellent results in vesiccation without producing permanent blemishes: ℞.—Unguentum basilica, 100 g.; thapsiæ, 5 g.—M. It can be preserved for a long time.—*Bull. de la Soc. Cent. de Méd Vét.*

Action of Alcohol upon the Gastric Juice. Introduced into the stomach, alcohol increases the flow of the gastric juice. This result is only partly due to its local action. Frouin and Molinier have obtained the same results with alcohol introduced into the rectum in dogs with a gastric fistula.

They conclude: Alcohol produces a hypersecretion of the gastric juice, which is not alone due to its local action, since it produces the same result by its direct introduction into the intestine; that this is due to a special action upon the nervous system, and may continue for several days, and even increase in intensity.—*Ann. de Méd. Vét.*

Vaginal Gestation. De Bruin, of Utrecht, is of the opinion that these cases are merely displacements from the uterus into the vagina, on the surface of which they produce, under special conditions, more or less pronounced alterations.

Strebel takes a different view, and cites an instance in a heifer. A soft mass resembling the fœtal membranes was protruding from the vulva. In the vagina was found a mass as large as a man's fist and adherent to the mucous membrane. The mass was withdrawn with slight traction, and on its surface were seen over a small area small proliferations adhering to the vagina, but no cotyledons. The sac contained a well-formed male fœtus. The uterine neck was firmly closed, and exploration of the right flank revealed a second uterine fœtus. The latter was born in a normal manner at the end of gestation.

Strebel does not believe that the fœtus was expelled from the uterus ; that it is not probable that this mass would have been tolerated in the vagina as a foreign body, and that the fœtal placenta would not have formed adhesions with the vaginal mucous membrane.—*Ann. de Méd. Vét.*

Cricotomy for Roaring. The effects from section of the cricoid have been denied because the spreading of the cricoid cartilage did not widen the laryngotracheal canal. Blanchard operated on fifteen cases. Four were entirely relieved, three much improved.

Garcin communicates his results of fifty cases as follows :

Two race-horses have won races after the operation, which they could not do before.

One steeplechaser, with complete result.

One work horse, with good success.

Two race-horses, with good results.

One work horse, with complete success.

One driving horse, with complete success.

One work horse, with a surprising result.

The evidence of different veterinarians seems to be contradictory.—*Rec. de Méd. Vét.*

Antirabic Treatment. At the Institute of Vienna 1166 cases received the inoculation treatment for rabies : Male adults, 466 (39.3 per cent.); female, 205 (17.3 per cent.) ; children, 514 (43.5 per cent.); 16 of these (1.45 per cent.) died. Six of these cases should be excluded, because the disease developed within fifteen days, the time required to establish immunity, thus giving an actual mortality of only 0.94 per cent. Wounds of the head and face and the hands were the most fatal.—*Oesterr. Monatsch. für Thierheilk.*

